# Analysis of effects of elevation on the power output and efficiency of ground-mounted photovoltaic modules

**DOI:** 10.1038/s41598-026-37413-1

**Published:** 2026-01-27

**Authors:** Aschenaki Tadesse Altaye, István Farkas, Piroska Víg

**Affiliations:** 1 Doctoral School of Mechanical Engineering, Hungarian University of Agriculture and Life Sciences, Gödöllő, Hungary; 2 Institute of Technology, Hungarian University of Agriculture and Life Sciences, Gödöllő, Hungary; 3 Institute of Mathematics and Basic Science, Hungarian University of Agriculture and Life Sciences, Gödöllő, Hungary; 4 Department of Mechanical Engineering, Hawassa University, IoT, Ethiopia

**Keywords:** ANOVA, Cell temperature, Electrical efficiency, Elevation height, Electrical power, Energy science and technology, Engineering

## Abstract

This study examines the effects of elevation on the performance of ground-mounted photovoltaic modules, focusing on power output and efficiency. Outdoor experiments were conducted to assess the influence of varying mounting elevations on the electrical performance of PV modules. An experimental setup was deployed at the Hungarian University of Agriculture and Life Sciences (MATE), where three identical polycrystalline PV modules were installed at elevations of 0.7 m, 1.1 m, and 1.6 m above a concrete surface. All modules were south-facing with a fixed 45° tilt to ensure consistent solar exposure. Measurements of solar irradiance, ambient and module temperatures, voltage, and current were recorded from 10:00 to 16:00 under clear-sky conditions. The PV module elevated at 1.1 m demonstrated the highest mean power output (31.64 W) and efficiency (6.67%), outperforming the modules at 0.7 m (25.34 W; 5.36%) and 1.6 m (19.70 W; 4.29%). Enhanced airflow and moderate albedo at 1.1 m reduced cell temperatures, improving electrical performance. Statistical analysis using ANOVA and Tukey’s HSD confirmed that elevation height significantly influenced both power and efficiency (*p* < 0.001). The results highlight that an elevation of approximately 1.1 m optimises convective cooling and irradiance capture, providing a cost-effective strategy to enhance PV energy yield and operational reliability. This system offers strong techno-economic and environmental viability, characterised by a $0.0843 kWh⁻¹ levelized cost of electricity, and a CO₂ mitigation of 577.78 kg over 25 years.

## Introduction

Climate change is an increasingly critical global challenge, exacerbating extreme weather events and necessitating a rapid transition from fossil fuels^1,2^. Consequently, growing awareness of climate change, coupled with the finite nature of fossil fuels and escalating global energy demands, has spurred a notable transition towards sustainable and renewable energy solutions^3,4^. Indeed, this energy transition represents a crucial step in mitigating the environmental consequences of fossil fuel reliance and curtailing greenhouse gas emissions^5^. To effectively facilitate this transition, a range of technologies has been developed, encompassing alternative fuels, solar, wind, and biomass energy, in conjunction with ongoing efforts to enhance the efficiency of associated equipment. PV arrays are particularly suitable for both urban and rural settings, making them a versatile option for energy generation^6^.

Solar irradiance generally correlates positively with power output; however, this relationship is moderated by temperature. Specifically^7^, observed that temperature effects become more pronounced at high irradiance levels. Elevated module temperatures inversely affect PV performance, reducing voltage and accelerating material degradation, which impacts long-term reliability; therefore, effective thermal management is crucial^8^. Studies address this need^9^ demonstrating enhanced convective cooling and improved energy output through optimised panel elevation and arrangement. Furthermore^10^, identified optimal tilt and azimuth angles in South Africa, emphasising thermal considerations.

In addition to solar radiation and temperature, soiling, caused by the accumulation of dust and other particulates on PV module surfaces, reduces light absorption and diminishes energy generation. The extent of this performance reduction depends on the nature and concentration of the soiling, as well as environmental conditions^11^^12^. modelling further elucidates this, showing that particle size influences dust deposition rates and, consequently, cleaning schedules. Wind presents a dual influence: while it can enhance cooling and improve efficiency, strong winds can also pose a risk of mechanical damage to modules and mounting structures. Moreover, wind contributes to soiling by carrying dust and debris^13^. Accordingly^14^, examined wind loads, highlighting the need for refined design protocols. Shading is another environmentally critical factor, as even partial shading can significantly decrease power output due to reverse biasing of shaded cells^15^.

Water exposure can negatively affect PV units, inducing corrosion, damaging components, and reducing electrical efficiency. High relative humidity exacerbates these effects, especially in warm and humid climates^16^. Beyond these direct environmental influences, system performance is also affected by installation parameters and location. Studies demonstrate that adjusting panel height, inclination angle, and mounting configurations can optimise performance. For example^17^, showed that lowering PV panel height on a green roof enhances output. Similarly^18^, found that adjusting panel height in Agri photovoltaic systems can improve thermal conditions^19^. found that floating PV systems benefit from enhanced cooling compared to ground-mounted ones in India. At the same time^20,21^, highlighted the advantages of rooftop systems, particularly in mitigating the effects of hot climates. The impact of location is further underlined by^22^, whose study of a desert PV farm in China revealed localised microclimate changes, stressing the importance of site-specific planning^23^. demonstrated that row spacing optimisation is crucial for maximising yield in bifacial PV systems.

To mitigate the negative effects of environmental factors and enhance PV performance, researchers have explored various optimisation and modelling techniques^24^. employed a genetic algorithm to optimise grid-connected PV designs, and^25^ compared simulation tools for optimising bifacial PV systems^26^. effectively used the Taguchi method to optimise bifacial PV module orientation in India, further highlighting the role of strategic installation parameters in performance. Finally^27^, studied agrivoltaic systems and shading management, highlighting their importance in mitigating the effects of urban heat islands.

Elevating photovoltaic modules above the ground demonstrably affects their energy yield, especially for bifacial panels. Increasing module height mitigates rear-side irradiance non-uniformity and enhances energy capture, leading to higher peak power and overall energy output when the elevation exceeds approximately 1 m^28^. Furthermore, the ground surface condition, specifically its reflectivity (albedo), strongly influences the amount of ground-reflected solar radiation incident on the modules. High-albedo surfaces, such as snow, white concrete, gravel, or engineered reflective materials, significantly enhance rear-side irradiance, thereby increasing bifacial gain and overall energy yield. Empirical and modelling studies consistently indicate that ground albedo and module elevation are critical determinants of bifacial photovoltaic system performance, making them essential site-specific parameters for PV system design^29^.

The economic assessment of photovoltaic (PV) systems identifies the key factors that govern their financial viability across different applications and geographic contexts. Prior studies consistently emphasise the importance of robust economic evaluation frameworks for accurately assessing PV investments^30^. Highlight the use of net present value (NPV) and discounted cash flow analysis (DCFA) to evaluate ground-mounted PV systems, identifying system configuration, installed capacity, energy demand, and grid connection costs as critical determinants of profitability. Similarly^31^, applies internal rate of return (IRR), levelized cost of energy (LCOE), and capacity factor (CF) to assess a solar power plant in Iran, demonstrating the significant influence of feed-in tariffs and policy incentives on investment attractiveness.

The role of policy frameworks, particularly feed-in tariffs (FiTs), emerges as a recurring theme in the literature^32^. Compare rooftop PV investments in Italy and Germany, showing that while Italy’s initially generous FiTs stimulated early profitability, subsequent policy revisions introduced uncertainty, whereas Germany’s gradual and market-oriented tariff structure enabled more stable economic performance. In the Hungarian context^33^, confirms that PV investments remain economically viable under existing regulations and recommends small-scale systems for cost-sensitive investors.

In addition to policy influences, system design and operational strategies significantly affect economic outcomes^34^. demonstrate that manually adjustable tilt mechanisms enhance NPV and reduce payback periods in regions with high solar potential^35^. further examine maintenance strategies for ground-mounted PV plants, highlighting how plant size, logistics, spare-parts management, and electricity market conditions influence cost-effectiveness. More recently^19^, compared floating and ground-mounted PV systems, reporting that floating PV achieves 6–7% higher power output due to lower operating temperatures (4–6 °C). Although ground-mounted systems generally offer lower LCOE and shorter payback periods, floating PV significantly reduces land use and contributes to the achievement of the UN Sustainable Development Goals^36^.

The objective of this study is to examine the effect of mounting elevation on the electrical performance of ground-mounted photovoltaic modules to determine the height that maximises energy yield.

*Research gap and novelty*.

Although extensive research has optimised PV performance through adjustments in tilt angle, azimuth, and row spacing, systematic investigations of module elevation height in conventional ground-mounted PV systems remain limited. Existing studies have examined elevation effects primarily within specific applications such as green roofs, agrivoltaic systems, and floating PV installations or as part of broader thermal management strategies, without explicitly treating elevation height as an independent design parameter.

This research presents a novel investigation into the influence of mounting elevation on photovoltaic (PV) module performance when deployed on concrete surfaces. A controlled field experiment, utilising matched polycrystalline modules at varying heights, was conducted to isolate the effects of module elevation above the concrete. Comprehensive, simultaneous measurements of environmental conditions and PV module performance metrics were acquired and subsequently analysed using ANOVA to determine the statistical significance of elevation-dependent variations in electrical output. This rigorous approach identifies the optimal elevation for maximising electrical conversion efficiency and operational reliability in concrete-mounted PV systems, thereby addressing a significant gap in the existing literature.

## Materials and methodology

In this study, we provide a thorough description of the materials utilised, including their specifications and pertinent properties. This level of detail ensures that other researchers can replicate the exact conditions under which the study was conducted. Additionally, we outline the experimental research design employed, along with comprehensive data collection procedures and a detailed account of the variables measured. The statistical or analytical methods applied in our analysis are also specified.

### Materials

To ensure accurate and comprehensive data acquisition, the materials detailed in Table 1 were carefully selected and utilised throughout the experimental measurements. Furthermore, dedicated computing systems were employed to facilitate data logger configuration and control, real-time monitoring of experimental parameters, and subsequent data processing, analysis, and visualisation. The judicious selection and integration of these materials and computing resources were fundamental to achieving the objectives of this study, enabling the precise and reliable measurement of key thermal and electrical parameters.

Table 1 details the materials used in this study, along with their respective measurement accuracies and functions.

**Table 1:** Experimental devices used in this investigation.

Devices	Measurement accuracy	Function	Figure
ALMEMO-Ahborn-2890/9	Depends on the digital ALMEMO-Ahlborn- 2890/9 sensors used	Recording data	
ALMEMO-ZA-9020-FS (K-type) and Denkovi	± 0.5 °C	Temperatures of: -ambient -PV surface and back cells	
MessKopF3.3 pyranometer	<±10 W/m^2^	Solar radiation	
DENKOVI-SMART-32channel	± 0.5% ±0.5%	Module voltage Module current	
1 ohm resistors		Voltage reduction	

### Methodology

**Fig. 1:**
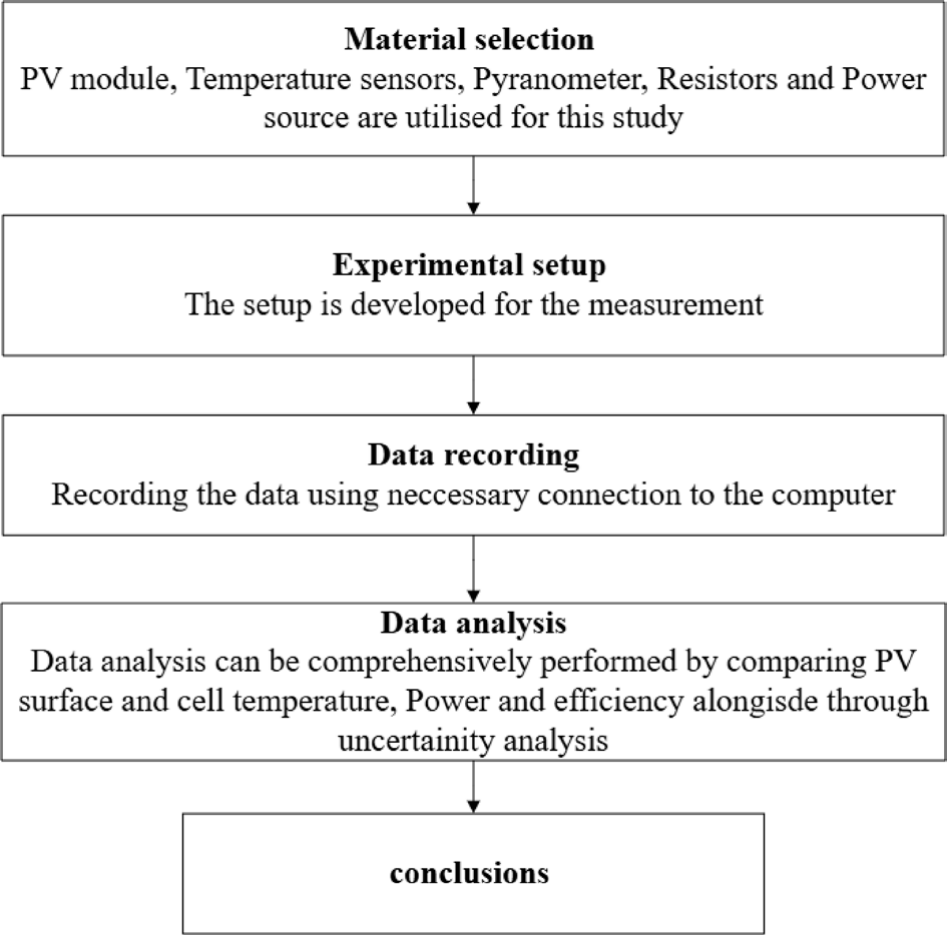
Workflow diagram.

Figure 1 presents a workflow diagram outlining the methodology employed in this study, encompassing material selection, experimental setup, data acquisition, and subsequent data analysis.

This study investigates the influence of varying elevation heights on the operational performance of ground-mounted photovoltaic (PV) modules. A controlled experimental setup was established, comprising three identical PV modules, each mounted at a fixed tilt angle of 45°, a configuration optimised for solar irradiance capture. All modules were installed in the same plane with a south-facing orientation to ensure uniform solar exposure. A reference pyranometer, aligned with the module tilt, measured incident solar radiation under clear-sky conditions. By maintaining consistent module specifications, including size, orientation, and inclination, this design isolates elevation as the primary variable, enabling a robust comparative analysis of how elevation-dependent environmental factors affect PV performance.

The experiment was conducted on 21 September 2025, with continuous measurements of solar irradiance, PV module surface and cell temperatures, and electrical output recorded from 10:00 to 16:00 local time, covering the primary daylight hours. The three-second sampling interval was chosen to capture short-term fluctuations in environmental and operational parameters, including solar irradiance, wind speed, module temperature, voltage, and current, under real outdoor conditions. These parameters are inherently dynamic and can vary rapidly due to passing clouds, wind gusts, and transient thermal effects. High-frequency sampling, therefore, ensures that such variations are accurately recorded and that no significant transient behaviour is overlooked.

To enhance data reliability and minimise measurement noise, the raw data collected at three-second intervals were averaged over a predefined time window of six minutes before analysis. This averaging process smooths short-term random fluctuations while preserving the underlying physical trends relevant to PV performance evaluation. The averaged values were then used for comparison across different elevations to ensure consistency and statistical robustness.

The data collection process was conducted in short time intervals, resulting in a comprehensive dataset that spans several hours. This meticulous approach allowed us to gather a substantial amount of information for analysis. To enhance clarity and facilitate interpretation, we focused on calculating the average values derived from this extensive dataset. The subsequent analysis provided valuable results that contribute significantly to our understanding of the observed phenomena during this time period.

**Fig. 2:**
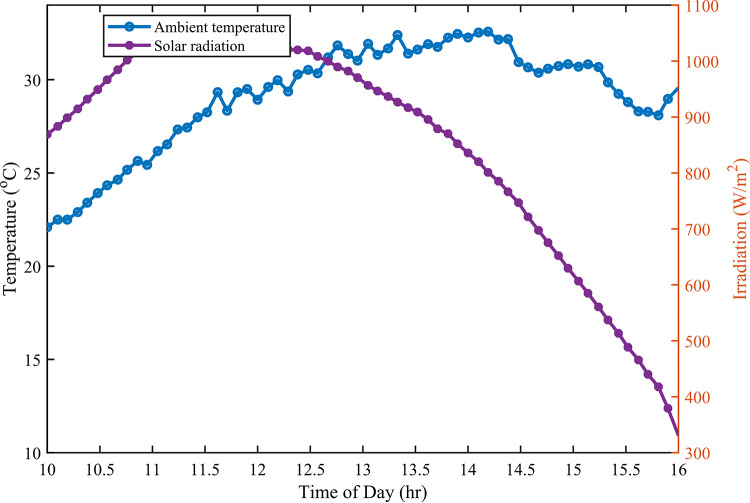
Solar radiation and ambient temperature input variables.

As illustrated in Fig. 2, the fluctuations in the input variables of solar radiation and ambient temperatures throughout the day are clearly depicted. The maximum recorded temperature reached an impressive 32.6 °C, coinciding with peak solar radiation levels of 1021.4 W/m^2^ at for a significant duration of the experimental work. This correlation highlights the relationship between elevated temperatures and increased solar energy availability during this time frame, providing valuable insights into environmental conditions affecting photovoltaic performance and energy generation potential.

**Fig. 3:**
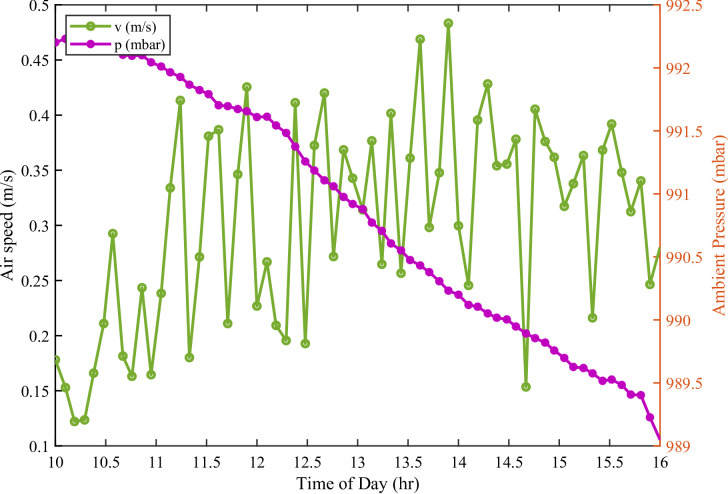
Input variables of air speed and ambient pressure recorded during investigation.

Figure 3 illustrates the variations of input variables like; ambient air speed (v, m/s) and atmospheric pressure (p, mbar), from 10:00 to 16:00. These variations demonstrte the dynamic atmospheric conditions influencing the PV modules during the experiment. Air speed generally increased towards midday. This increase was likely due to solar-driven convection. The increased air speed enhanced forced convective cooling of the module surfaces. Higher wind velocities improved heat dissipation. This helped limit PV cell temperature rise and sustained electrical efficiency. Atmospheric pressure exhibited moderate variations. These variations had a minor effect on air density. Consequently, the effect on cooling capacity was also minor compared to wind speed. Overall, the observations highlight the dominant role of ambient airflow. This airflow regulates PV module temperature and performance under outdoor conditions, relative to atmospheric pressure.

### Experimental setup

**Fig. 4:**
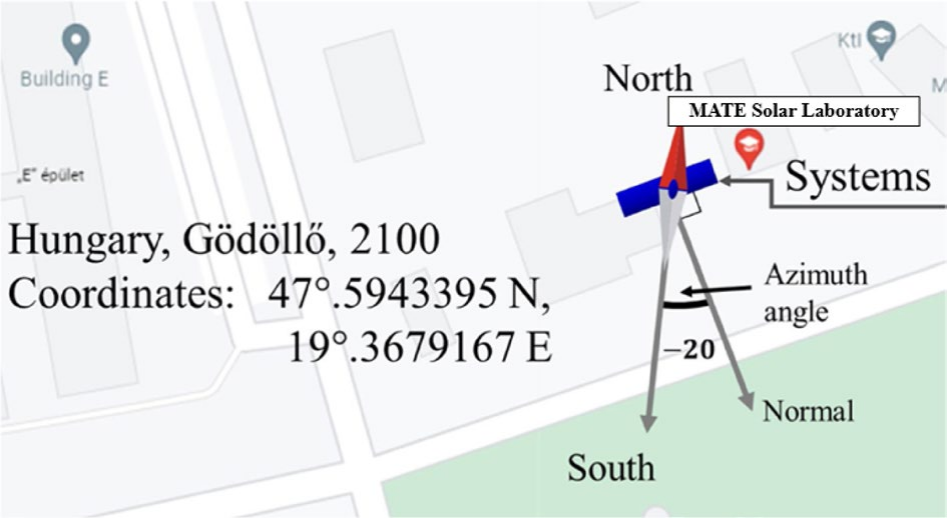
Geographical location of the conducted experiment^37^.

This study utilised an accurately designed experimental setup to determine the optimal elevation for maximising energy yield and enhancing overall photovoltaic system performance. To ensure accurate and comprehensive data acquisition, a suite of precisely calibrated instruments was employed. Specifically, calibrated temperature sensors were strategically positioned at critical locations to capture temperature variations, providing essential data for reliable thermal characterisation. Global solar irradiance, a key parameter in this investigation, was measured using pyranometers to quantify solar radiation incident on a PV module surface. Polycrystalline PV modules served as the subject of the investigation, and their electrical performance was analysed through the integration of precisely calibrated resistors into the electrical circuits, enabling controlled manipulation and measurement of current and voltage parameters. To capture the dynamic behaviour of the system, programmable Denkovi data loggers were used for the automated and continuous recording of electrical data, including PV module outputs and voltage drops across the resistors. Concurrently, a high-precision, multi-channel Almemo data logger facilitated the synchronised acquisition of data from the solar radiation sensors. These measurements were conducted in the study areas, as depicted in Fig. 4, which provides a comprehensive overview of the experimental study area utilised in this research.

**Fig. 5:**
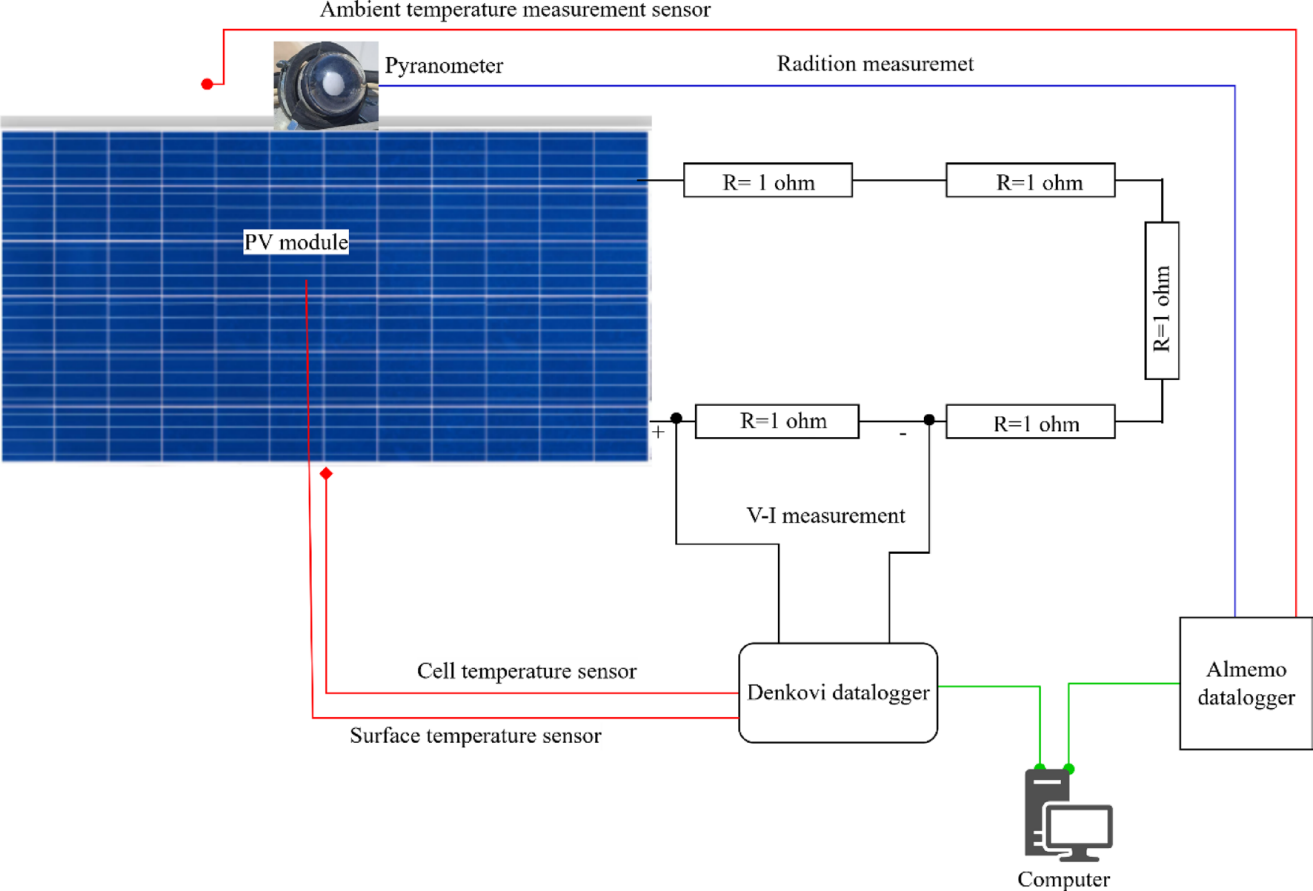
Schematic diagram of experimental setup.

Figure 5 illustrates the experimental setup designed to assess the electrical and thermal performance of the photovoltaic (PV) module. The module was connected to a resistive load network comprising five 1 Ω resistors, allowing for controlled loading and precise voltage and current measurements. Internal cell temperatures were monitored using strategically placed temperature sensors to characterise the module’s thermal behaviour.

All sensors were connected to a Denkovi data logger, which relayed both electrical and thermal data to an Almemo data logger and subsequently to a dedicated computer. This configuration enabled real-time monitoring and synchronised acquisition of performance parameters, ensuring data consistency and reliability throughout the experiment. The integrated system facilitated detailed post-processing, enabling accurate correlation between module temperature and electrical output at varying conditions.

Solar Radiation Measurement: Global solar irradiance (W/m²) incident on the PV module was measured using a pyranometer, capturing both direct and diffuse components. The pyranometer’s output was recorded by an Almemo data logger, which accommodates multiple analog inputs.

Temperature Monitoring: Temperature sensors, positioned at key locations on and near the PV module, recorded internal cell temperatures. These readings were logged via the Denkovi system to maintain synchronisation with other environmental and electrical parameters.

Electrical Characterisation: The module’s electrical characteristics, including current–voltage behaviour, were accurately measured using a resistive load bank. Voltage and current outputs were captured by Denkovi data loggers equipped with appropriate analog inputs.

Centralised Data Management: Data streams from both Almemo and Denkovi loggers were transmitted to computer systems for unified storage and processing. These systems managed logger configuration, real-time monitoring, and comprehensive data analysis.

Prior to use, all sensors were calibrated according to the manufacturer’s specifications. Data acquisition was performed at 3-second intervals to ensure sufficient temporal resolution for capturing transient thermal and electrical phenomena.

**Fig. 6:**
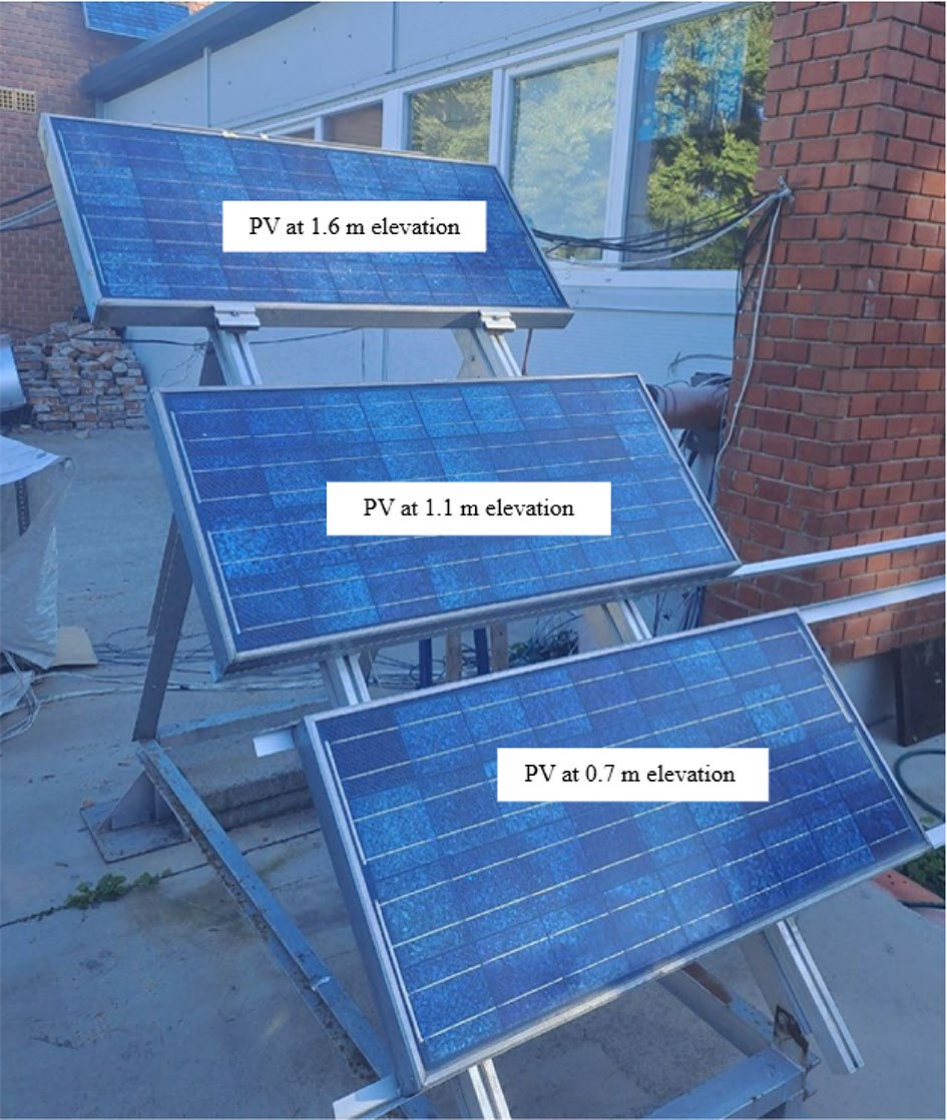
Photograph of the experimental rig.

Figure 6 illustrates the actual experimental setup utilised during data measurements, providing a clear overview of how the experiments were conducted. This visual representation aids in understanding the configuration and arrangement of equipment used throughout the study.

Table 2 presents the key performance characteristics of the photovoltaic module selected for this study, as specified under standard test conditions (STC).

**Table 2:** Specification of the PV module used in this experiment.

Parameters	Value
Voltage at maximum power (V_mp_)	17.1 [V]
Current at maximum power (I_mp_)	3.5 [A]
Maximum power (P_max_)	60 [W]
Short-circuit current (I_sc_)	3.8 [A]
Open circuit voltage (V_oc_)	21.1 [V]
PV module area (A_m_)	0.55 [m^2^]
Temperature coefficient of power	(0.5 *±* 0.05) [%/^o^C]

### Measurement data analysis

The performance of the photovoltaic (PV) modules was rigorously evaluated and compared using a specific equation designed to quantify their efficiency and power output under varying operational and environmental conditions. This approach allowed for a precise understanding of how effectively the modules convert solar energy into electricity.

Electrical power of the selected module can be evaluated using the following Eqs^38,39^. :

1 $$\:{P}_{el}=V\:I$$

Electrical efficiency of the selected module is defined by^40^:

2 $$\:{\eta\:}_{el}=\frac{{P}_{el}}{G{A}_{m}}$$

## Result and discussion

The experimental investigation, as depicted in Fig. 4, was executed at the MATE campus in Gödöllő city, situated in the central region of Hungary. The PV module was installed on a concrete ground surface. Throughout the experimental period, key parameters were meticulously recorded, encompassing solar irradiance, ambient temperature, photovoltaic (PV) cell temperature, PV module surface temperature, voltage, and current. The subsequent sections detail the findings of this study, presenting the data acquired from these measurements.

The albedo effect, or ground-reflected radiation, can enhance irradiance on the rear and surrounding areas of a PV module. Concrete surfaces, commonly used for PV module installations, reflect a substantial amount of radiation. This reflected radiation can strike the PV module, particularly its backside, thereby increasing the temperature, especially when the module is close to the ground.

**Fig. 7:**
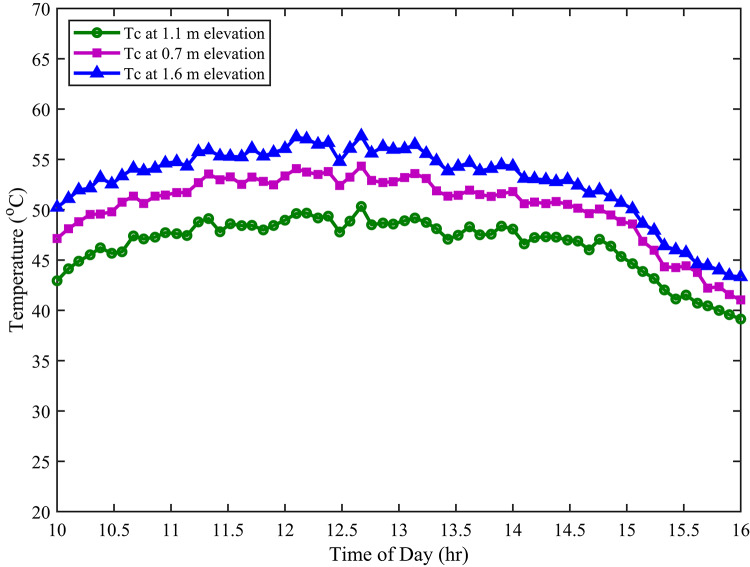
PV cell temperature at different elevations.

Figure 7 presents a comparison of PV cell temperatures at different elevations. The investigation indicates that the cell temperature (back side temperature) of the PV module at 1.1 m elevation is lower than that at 0.7 m and 1.6 m. This temperature reduction can be attributed to the airflow patterns at an elevation of 1.1 m. Specifically, moderate airflow at this medium elevation likely enhances convective cooling of the PV cells. Convective heat transfer is directly related to air velocity; an optimal airflow rate removes heat more effectively than stagnant or excessively turbulent conditions. At lower elevations (0.7 m), airflow may be restricted due to proximity to the ground and albedo effects, resulting in reduced cooling. Conversely, at higher elevations (1.6 m), increased turbulence could disrupt the laminar boundary layer on the PV module surface, reducing the effectiveness of convective cooling.

The observed temperature difference leads to a significant operational advantage. 1.1 m elevated PV cell temperatures are known to improve both efficiency and overall performance. According to the Shockley-Queisser limit, the band gap energy of a semiconductor material, and thus its efficiency in converting photons to electricity, is temperature-dependent. Elevated temperatures increase the intrinsic carrier concentration and reduce the open-circuit voltage, thereby decreasing the cell’s efficiency. Therefore, maintaining lower operating temperatures through optimised airflow, as observed at the 1.1 m elevation, is crucial for maximising PV system performance.

Furthermore, to record voltage and current, we connect the terminals of one of the resistors to the data logger to record low voltage values that do not exceed 10 volts. According to Ohm’s law (V = RI), with a resistance of 1 ohm, the value of voltage will be equal to the current (V = I). This relationship allows us to determine the current value directly.

**Fig. 8:**
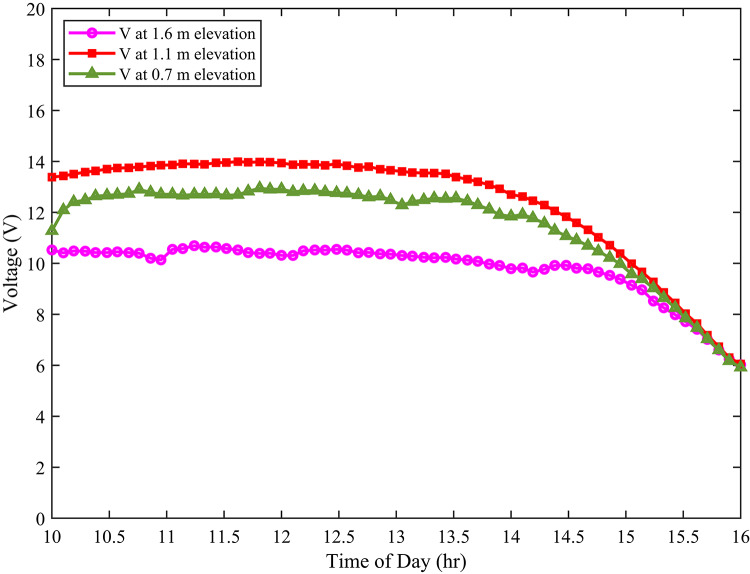
Module output voltage at different elevations.

As a result, variation of cell temperature, Fig. 8 illustrates the voltage output at different elevations. The voltage at 1.1 m elevation is higher compared to those at 0.7 m and 1.6 m. This difference is due to the combined effect of moderate albedo and airflow conditions at this elevation.

Albedo refers to the fraction of solar radiation reflected by the surface. A moderate albedo can optimise the amount of solar radiation incident on the PV module. Too little reflected radiation reduces the overall light available for conversion. Too much reflected radiation can increase the module’s temperature, negatively impacting voltage output. The 1.1 m elevation likely provides a balanced albedo effect. Moreover, airflow also plays a crucial role. As previously discussed, moderate airflow enhances convective cooling. Lower temperatures improve the voltage output of PV cells. This is because the open-circuit voltage of a solar cell has a negative temperature coefficient.

**Fig. 9:**
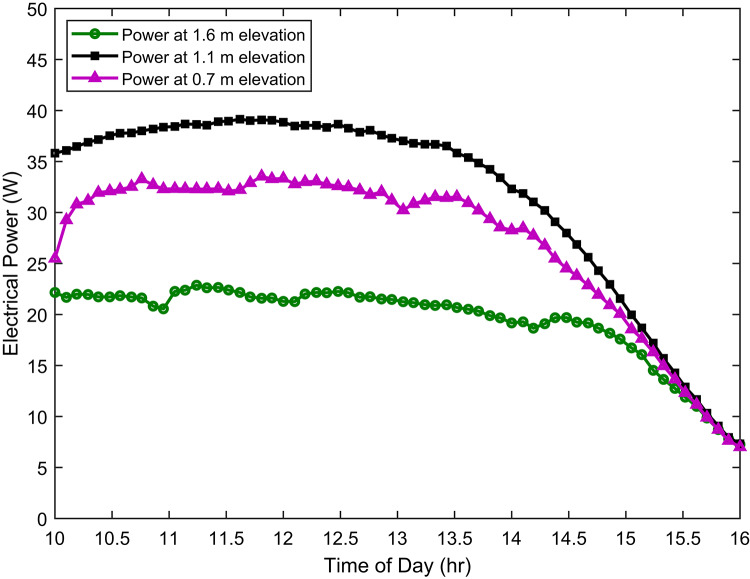
Electrical power generation at different elevations.

Building upon previous observations regarding temperature, airflow and albedo effects, Fig. 9 presents a detailed analysis of the electrical power generation from ground-mounted photovoltaic (PV) modules at varying elevations. The electrical power output is a direct consequence of the interplay between these environmental factors.

Specifically, the optimal balance of conditions observed at the 1.1 m elevation, namely, moderate airflow for effective cooling, balanced albedo for optimised solar irradiance, translates directly into enhanced electrical power generation. As previously discussed, moderate airflow reduces PV cell temperature. Lower temperatures increase cell voltage and efficiency. Balanced albedo ensures sufficient solar radiation without excessive heat buildup.

Conversely, the lower power generation observed at 0.7 m and 1.6 m can be attributed to suboptimal conditions. At 0.7 m, reduced airflow and increased albedo effects limit power production. At 1.6 m, potentially turbulent airflow and variations in albedo may negatively impact performance. Therefore, this investigation underscores the importance of considering microclimatic factors in PV system design and placement to enhance overall performance and energy yield.

**Fig. 10:**
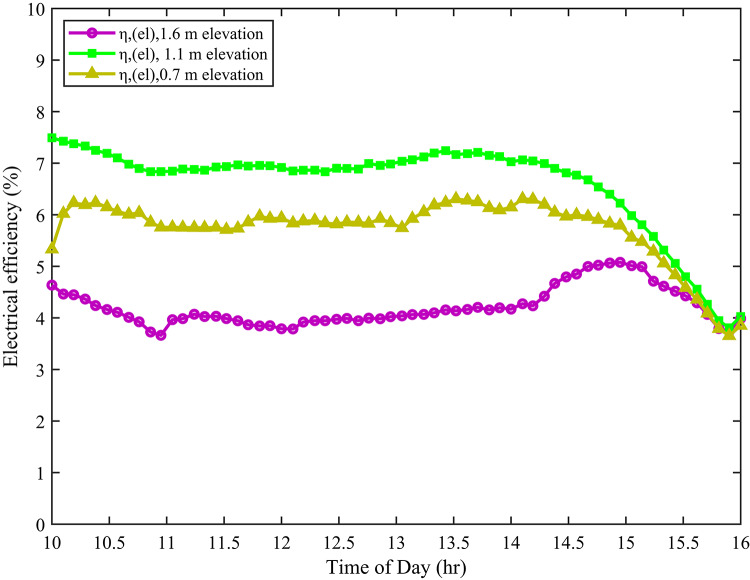
Electrical efficiency at different elevations.

The hourly efficiency trend of photovoltaic modules exhibits a characteristic diurnal pattern driven by fluctuations in solar irradiance and thermal conditions. As depicted in Fig. 10, electrical efficiency generally peaks during the midday hours, with a notable reduction observed around late afternoon, specifically at 14:00. This rise coincides with the period of maximum solar radiation and ambient temperatures. Although increased irradiance has the potential to enhance energy conversion, elevated module temperatures, particularly around midday, lead to thermal losses due to the temperature-dependent properties of semiconductor materials. Furthermore, comparative analysis across different installation heights indicates that the module positioned at 1.1 m consistently achieves higher efficiency values. This outcome can be attributed to enhanced convective heat dissipation at moderate elevations, which helps to maintain lower cell temperatures and improve performance. These findings underscore the critical role of both environmental factors and installation configuration in optimising PV system efficiency throughout the day.

In general, the measurements of this study indicate that the PV module at a mounting elevation of 1.1 m consistently generated a higher power output, approaching 39.1 W. This finding is notable considering the observed cell temperatures. Although the cell temperature at 1.1 m was more favourable (lower, correlating with improved efficiency), the power output remained superior. This suggests that while lower cell temperatures are generally beneficial, other factors at the 1.1 m elevation may contribute to enhanced power generation. These factors include airflow and albedo.

At the lowest elevation of 0.7 m, ground proximity partially restricts airflow around the PV module, resulting in diminished convective cooling and elevated operating temperatures. Conversely, the highest elevation of 1.6 m, while benefiting from stronger wind flow, is also subjected to environmental fluctuations, potentially reducing the stability of heat dissipation and electrical performance under real outdoor conditions. The 1.1 m elevation provides sufficient wind-induced convective cooling without excessive turbulence. This leads to lower and more stable module operating temperatures, consequently enhancing PV electrical efficiency and power output. Therefore, wind speed is a critical factor in determining the maximum PV power output and efficiency.

To further maximise electrical power generation at this optimal 1.1 m elevation, implementing cooling mechanisms for the PV cells is recommended. These interventions are crucial for mitigating thermal losses, which can otherwise decrease efficiency and energy output. Effective cooling strategies, such as water spraying, forced air cooling, nanofluids, and phase change materials, are recommended to reduce panel temperatures and boost efficiency. These methods dissipate heat through both conduction and convection.

The cell temperature at a height of 1.1 m exhibited a notable reduction compared to other elevations. Specifically, it was approximately 4–5 °C lower than the temperature observed at 0.7 m and 7–9 °C lower than at 1.6 m. Furthermore, this thermal advantage translated into improved electrical performance. Indeed, the module at 1.1 m generated about 4–5 W more electrical power than the module at 0.7 m. Moreover, it outperformed the module at 1.6 m by 15–16 W. In addition to power output, efficiency also benefited from the 1.1 m elevation. As a result of superior thermal stability and irradiance conditions, the efficiency at 1.1 m was approximately 1–2% higher than at 0.7 m. Similarly, in comparison to 1.6 m, the efficiency improvement was around 2–4%.

### Uncertainty analysis

As detailed in Table 1, the inherent uncertainties associated with the sensors employed in this study may introduce measurement errors during experimentation. To quantify and address these potential errors, Eqs. 3 and 4 were utilised to determine the overall uncertainty in the measurements^41^:

3 $$\:{\delta\:}_{p}=\sqrt{{{\left(\frac{{\delta\:}_{V}}{{V}_{1}}\right)}^{2}{\left({P}_{1}\right)}^{2}+\left(\frac{{\delta\:}_{I}}{{I}_{1}}\right)}^{2}{\left({P}_{1}\right)}^{2}+\dots\:+{{\left(\frac{{\delta\:}_{V}}{{V}_{n}}\right)}^{2}{\left({P}_{n}\right)}^{2}+\left(\frac{{\delta\:}_{I}}{{I}_{n}}\right)}^{2}{\left({P}_{n}\right)}^{2}}$$

4 $$\:{\delta\:}_{\eta\:}=\sqrt{{{\left(\frac{{\delta\:}_{P}}{{P}_{1}}\right)}^{2}{\left({\eta\:}_{1}\right)}^{2}+\left(\frac{{\delta\:}_{P}}{{A}_{m}*G}\right)}^{2}{\left({\eta\:}_{1}\right)}^{2}+\dots\:+{{\left(\frac{\delta\:p}{{P}_{n}}\right)}^{2}{\left({\eta\:}_{n}\right)}^{2}+\left(\frac{\delta\:p}{{A}_{m}*G}\right)}^{2}{\left({\eta\:}_{n}\right)}^{2}}$$

where, $\:{\delta\:}_{V},{\delta\:}_{I\:}and\:{\delta\:}_{P}$are represents the voltage, current and power and efficiency for which uncertainty is being evaluated, while *n* denotes the variables upon which the parameter *P* and $\:\eta\:$ are dependent. In this study, we conducted an uncertainty analysis for two key performance metrics: electrical power output and efficiency. This analysis helps to quantify the degree of uncertainty associated with our measurements and calculations, providing a clearer understanding of their reliability.

The specific uncertainty values determined for each parameter, power and efficiency are calculated using Eqs. 3 and 4, resulting in values of 1% and 1.4%, respectively. These results from the uncertainty analysis indicate the extent to which a measured value is free from error. Furthermore, a smaller uncertainty typically signifies a higher level of precision in the measurement. In this study, both electrical power output and efficiency demonstrate lower uncertainty values, suggesting that these measurements are more precise due to the accuracy of the measurement devices used in data recording.

Moreover, since uncertainty often scales with the measured value, as indicated in Eqs. 3 and 4, it is directly proportional to power. Consequently, the higher power observed at an elevation of 1.1 m naturally results in a larger absolute uncertainty, even if the relative uncertainty remains constant.

### Statistical analysis

Analysis of variance (ANOVA) is widely employed to determine whether differences in mean PV performance metrics across measurements are statistically significant^42^. When ANOVA results indicate significance, Tukey’s Honest Significant Difference (HSD) test can further identify which specific groups differ, while controlling for family-wise error rates^43^. Moreover, the use of error bars in graphical representations provides a visual understanding of measurement variability, aiding in the interpretation of results.

*ANOVA Input Parameters*:

- Independent Variable (Factor): Elevation, treated as a categorical variable with multiple defined elevation levels.
- Dependent Variables (Responses): Continuous performance metrics measured at each elevation level, including power output and electrical efficiency.

This study applies a comprehensive statistical framework combining ANOVA, Tukey’s post hoc test, and error bar analysis to systematically evaluate PV module power and efficiency. Such an approach ensures that performance differences are both statistically validated and practically interpretable, contributing to optimised PV system design and deployment.

In this study, a one-way analysis of variance (ANOVA) was applied to investigate the effect of elevation height (0.7 m, 1.1 m, and 1.6 m) on the electrical power output of PV modules. The summary statistics. Table 3 shows that the mean power outputs were 25.34 W at 0.7 m, 31.64 W at 1.1 m, and 19.70 W at 1.6 m. Variance values also differed across groups, with the highest variability observed at 1.1 m (81.72) and the lowest at 1.6 m (13.85), indicating possible differences in consistency of performance across elevations.

From a physical perspective, the results align with existing literature indicating that module elevation affects convective cooling and incident irradiance uniformity, both of which contribute to variations in power output^44,45^. The higher mean power observed at 1.1 m suggests that this elevation provides an optimal balance between cooling and irradiance capture, whereas excessive height (1.6 m) leads to reduced performance due to environmental influences such as increased wind turbulence.

**Table 3:** Statistical summary of mean power output.

ANOVA: Single Factor (elevation)						
SUMMARY						
Groups	Count	Sum [W]	Average [W]	Variance [W]		
Power,0.7 m	73	1849.84	25.34	76.93		
Power,1.1 m	73	2309.49	31.64	81.72		
Power,1.6 m	73	1438.37	19.7	13.85		

ANOVA						
Source of Variation	SS	Df	MS	F	P-value	F crit
Between Groups	5202.92	2	2601.46	45.24	3.89E-17	4.7
Within Groups	12420.7	216	57.5			
Total	17623.6	218				

Similarly, Table 4 reveals a highly significant effect of elevation height on PV efficiency (F = 134.21, *p* = 1.31 × 10–^38^. The calculated F-value is substantially greater than the critical value (F_crit_=3.71) at a 95% confidence level, confirming that at least one elevation group mean differs significantly from the others. The extremely small p-value (< 0.001) indicates that the observed differences in mean efficiency are not due to random variation, but rather reflect a true effect of elevation height. From a physical perspective, the results can be explained by the combined effects of thermal regulation and irradiance capture. At 1.1 m elevation, modules exhibited the highest average efficiency (6.67%), suggesting an optimal balance between improved convective cooling and uniform irradiance exposure. At lower elevation (0.7 m), reduced airflow and albedo likely lead to higher operating temperatures, which negatively affect efficiency due to the temperature coefficient of PV modules^46^. Conversely, at a higher elevation (1.6 m), although convective cooling may be enhanced, efficiency decreased (4.29%) due to increased wind turbulence or non-uniform irradiance distribution across the module surface^45^.

**Table 4:** ANOVA mean PV efficiency statistical summary.

ANOVA: Single Factor (elevation)						
SUMMARY						
Groups	Count	Sum	Average	Variance		
Efficiency,0.7 m	73	391.00	5.35	1.38		
Efficiency,1.1 m	73	486.95	6.67	0.76		
Efficiency,1.6 m	73	313.40	4.29	0.17		

ANOVA						
Source of Variation	SS	df	MS	F	*P*-value	F crit
Between Groups	207.07	2	103.54	134.20	1.31E-38	3.71
Within Groups	166.64	216	0.77			
Total	373.72	218				

*Post Hoc Analysis – Tukey’s HSD Test*

Following the one-way ANOVA results, which confirmed that elevation height significantly affects both power and efficiency of PV modules, a Tukey’s Honest Significant Difference (HSD) test was applied to perform pairwise comparisons between groups. The Tukey HSD is a robust post hoc method that controls the family-wise error rate when making multiple comparisons, ensuring that statistical significance is not overstated^44^.

The HSD statistic is calculated as:

5 $$\:HDS={q}_{crit}*\sqrt{MS/n}$$

6 $$\:ME={t}_{crit}*SE$$

7 $$\:SE=\sqrt{\frac{Variance}{n}}$$

where $\:{q}_{crit}$ and $\:{t}_{crit}$ are determined as 3.338 and 1.993, respectively.

MS_within_​ is the mean square within groups, and n is the sample size per group.

A Tukey Honestly Significant Difference (HSD) test was applied to assess the effect of module elevation on power output and conversion efficiency. According to the Tukey criterion, an absolute mean difference exceeding the calculated HSD value indicates statistical significance at the 95% confidence level.

As presented in Table 5, all pairwise comparisons yielded mean differences greater than their respective HSD thresholds (2.96 W for power and 0.34% for efficiency), confirming that elevation had a statistically significant impact on both parameters. Elevating the PV module from 0.7 m to 1.1 m and 1.6 m above the ground led to substantial changes in power output (maximum difference: 11.93 W) and efficiency (maximum difference: 2.38%).

These results highlight that module elevation plays a crucial role in PV performance by influencing thermal behaviour. Higher elevations enhance convective cooling and reduce operating temperatures, thereby improving electrical efficiency, whereas lower elevations restrict airflow beneath the module, increasing thermal stress and reducing overall performance.

**Table 5:** HDS test result for power and efficiency.

Power/Efficiency	Mean	Variance	SE	t-crit	ME	MS within	q crit	HSD	Pairwise Comparison	Significant?
Power, 0.7 m	25.34	76.93	1.03	1.99	2.05	57.5	3.34	2.96	6.30	Yes
Power, 1.1 m	31.64	81.72	1.06	1.99	2.11	57.5	3.34	2.96	5.64	Yes
Power, 1.6 m	19.7	13.85	0.44	1.99	0.87	57.5	3.34	2.96	11.93	Yes
Efficiency, 0.7 m	5.36	1.38	0.14	1.99	0.27	0.77	3.34	0.34	1.31	Yes
Efficiency, 1.1 m	6.67	0.76	0.1	1.99	0.2	0.77	3.34	0.34	1.06	Yes
Efficiency, 1.6 m	4.29	0.17	0.05	1.99	0.1	0.77	3.34	0.34	2.38	Yes

**Fig. 11:**
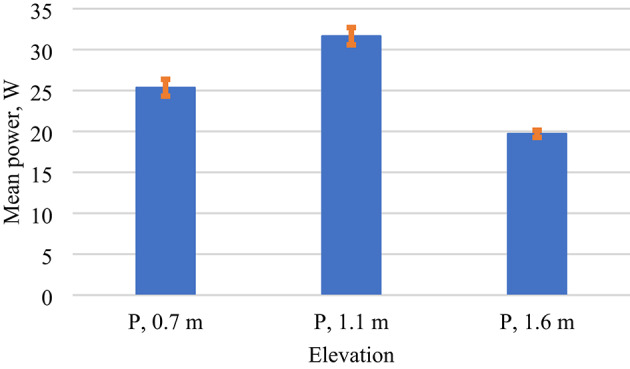
Mean power output at different elevations with standard error bars.

Figure 11. Mean power output (W) of photovoltaic modules recorded at three elevation heights: 0.7 m, 1.1 m, and 1.6 m. The vertical error bars represent the standard error of the mean (SEM), with values of 1.03 W, 1.06 W, and 0.44 W for the respective elevations (*n* = 73). These error estimates reflect the variability of the measurements and indicate the precision of the reported means.

**Fig. 12:**
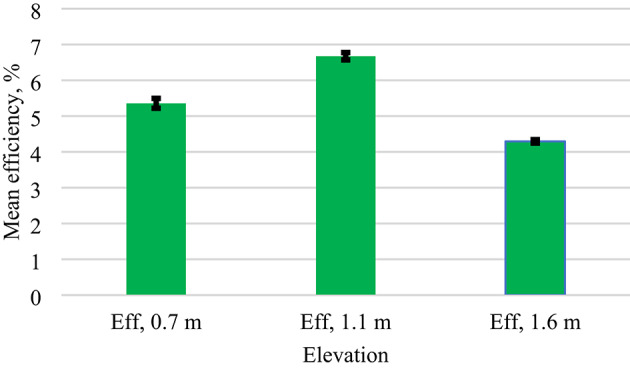
Mean efficiency at different elevations with standard error bars.

Similarly, Fig. 12. Mean efficiency (%) of photovoltaic modules at elevations of 0.7 m, 1.1 m, and 1.6 m. Error bars represent the standard error of the mean (SEM; 0.14%, 0.10%, and 0.05%, respectively; *n* = 73), indicating the precision of the measurements.

Finally, the combined ANOVA and Tukey HSD analyses demonstrate that elevation height is a critical design parameter affecting both the power and efficiency of PV modules. The 1.1 m elevation consistently outperformed other heights, suggesting it as the most effective mounting configuration under the tested conditions. Importantly, the statistical rigor of ANOVA and Tukey HSD ensures that these differences are not due to random variation but represent true, reproducible effects.

From a practical perspective, these results highlight the importance of elevation optimisation in PV system design. Proper elevation selection can improve energy yield and reduce thermal stress, ultimately contributing to enhanced long-term system reliability and economic performance.

The assumptions of normality and equal variances, required for ANOVA, are justified by the large sample size (*n* = 73 per elevation), the consistent and high-frequency data collection, and the uniform experimental conditions across all elevation levels. The continuous and smooth nature of the environmental variables, such as irradiance, temperature, and airflow, naturally promotes normally distributed performance responses, particularly after averaging. Furthermore, the recorded variances for power output and efficiency were comparable across groups, indicating no substantial heteroscedasticity. Collectively, these factors provide a sound statistical basis for applying ANOVA to evaluate the effect of elevation on PV performance.

### Economic and environmental impact assessment

Considering the economic implications of photovoltaic (PV) installations on system performance, profitability is determined by generation costs and production intensity^47^. While other energy systems may offer advantages, economic evaluations, particularly using metrics like levelized cost of electricity (LCOE), net present value (NPV), and payback time (PBT), are paramount for decision-makers. These assessments guide the prioritisation of energy technologies to ensure governmental profitability, reflecting a critical focus in contemporary research^48^.

Equations (8)– (11) are employed to assess the economic and environmental impacts of the photovoltaic (PV) system.

8 $$\:LCOE=\frac{NPV\:of\:total\:cost}{NPV\:of\:total\:energy\:produced}$$

9 $$\:LCOE=\frac{\sum\:_{n=0}^{N}\frac{{CC}_{n}+{PC}_{n}}{{(1+i)}^{n}}}{\sum\:_{n=0}^{N}\frac{{E}_{PV}({1-d)}^{n}}{{(1+i)}^{n}}}$$

10 $$\:NPV=\sum\:_{n=1}^{N}\frac{{NCF}_{n}}{{(1+i)}^{n}}$$

11 $$\:{co}_{2,n}=\sum\:_{n=0}^{N}\frac{{E}_{PV}({1-d)}^{n}}{{(1+i)}^{n}}*EF$$

where LCOE: Levelized cost of electricity, [$/kWh].

PCn: periodical cost [$].

CCn: Initial cost as the capital amount, [$].

E_PV_: Energy production in the first year, [kWh].

NPV: The Net Present Value, [$].

NCF: Net cash flow, [$].

PBT: Payback time, [year]

d: Degradation rate.

i: Real discount rate.

CO_2,n_ = CO₂ emissions avoided in year n [kg CO₂/year].

EF = grid emission factor [kg CO₂/kWh].

Table 6 presents the critical financial parameters underpinning the economic viability assessment. It concisely summarises the key financial assumptions and parameters employed in the economic analysis.

**Table 6:** Financial parameters for economic Assessment^47^^48^.

PV installed capacity (Wp)	60 Wp
Life time (N)	25
Real discount (r)	0.1
Degradation rate (d)	0.005
Peak sun hours (Hs)	5 h/day
Electricity price	0.12 ($/kWh)
Module cost	0.6 $/Wp
Electrical component price	0.2 $/Wp
Supplementary costs	0.23 $/Wp
Balance of system (BOS)	0.13 $/Wp
Operation and Management price	0.026 $/Wp/year

This assessment indicates that a ground-mounted photovoltaic (PV) system offers competitive life-cycle performance. An initial capital cost of $66.60, escalating to a total net present cost of $80.76 with discounted operation and maintenance, confirms cost efficiency, attributable to the low balance-of-system and maintenance demands typical of small-scale installations.

This system offers strong techno-economic and environmental viability, characterised by a $0.0843 kWh⁻¹ levelized cost of electricity, and a CO₂ mitigation of 577.78 kg over 25 years.

## Conclusion

This study systematically investigated the effect of elevation height on the electrical performance of ground-mounted photovoltaic (PV) modules under real outdoor conditions. Through a controlled experimental setup, high-resolution data acquisition, and rigorous statistical analysis, the following key findings were obtained:

- The PV module mounted at 1.1 m elevation consistently achieved superior performance, with a peak power output of 39.1 W and a mean efficiency of 6.67%, outperforming modules at 0.7 m and 1.6 m elevations.
- Lower PV cell temperatures minimised thermal losses associated with the negative temperature coefficient of PV modules, thereby sustaining higher voltage output.
- Albedo from the concrete surface increased rear-side irradiance but, when excessive at lower elevation, contributed to overheating.
- ANOVA and Tukey’s HSD tests confirmed that differences in power and efficiency across elevations were statistically significant (*p* < 0.001), with pairwise mean differences exceeding critical thresholds.
- In this study area, mounting PV modules at an intermediate elevation (1.1 m) can substantially enhance energy yield, improve thermal management, and prolong system reliability.
- This study illustrates robust techno-economic and environmental viability, indicated by a levelized cost of electricity (LCOE) of $0.0843 per kWh, and a CO₂ mitigation of 577.78 kg over 25 years.
- **Limitations and future work**.
- The study’s short-term data acquisition limits the assessment of seasonal, long-term weather, dust accumulation, and degradation impacts on photovoltaic performance.
- The investigation was restricted to three discrete elevation heights (0.7 m, 1.1 m, and 1.6 m), potentially overlooking optimal configurations outside this range.
- The findings are specific to concrete surfaces; the influence of alternative ground materials, such as vegetation, on irradiance reflection and thermal behaviour remains unexplored.
- Future studies should evaluate the long-term seasonal effects of elevation and microclimatic factors on PV performance.
- Investigating hybrid cooling solutions combined with optimal elevation can provide further insights into maximising efficiency and sustainability of PV systems.

## Data Availability

The data of this manuscript is available on request from the corresponding author.

## Abbreviations

CF: Capacity factor
Df: Degrees of freedom
FiTs: Feed-in tariffs
FPV: Floating photovoltaic
HSD: Honest significant difference
IRR: Internal rate of return
LCOE: Levelized cost of energy
ME: Margin of error
MS: Mean square
NPV: Net present value
p-value: Probability value
PV: Photovoltaic
SE: Standard error
SS: Sum of squares
STC: Standard test condition
Am: Module area (m^2^)
G: Solar radiation (W/m^2^)
H: Height (m)
I: Current (A)
Imp: Current at maximum power (A)
Isc: Short circuit current (A)
Pmax: Maximum power (W)
Pel: Electrical power (W)
R: Resistor (Ω)
Ta: Ambient temperature (°C)
Tc: PV backside cell temperature (°C)
Ts: PV top surface temperature (°C)
V: Voltage (V)
Vmp: Voltage at maximum power (V)
Voc: Open circuit voltage (V)
$$\:{\eta\:}_{el}$$: Electrical efficiency (%)
